# Integrated Multi-Omics Reveals Significant Roles of Non-Additively Expressed Small RNAs in Heterosis for Maize Plant Height

**DOI:** 10.3390/ijms24119150

**Published:** 2023-05-23

**Authors:** Jie Zhang, Yuxin Xie, Hongwei Zhang, Cheng He, Xiaoli Wang, Yu Cui, Yanfang Heng, Yingchao Lin, Riliang Gu, Jianhua Wang, Junjie Fu

**Affiliations:** 1 Center of Seed Science and Technology, Beijing Innovation Center for Seed Technology (MOA), Beijing Key Laboratory of Crop Genetic Improvement, College of Agronomy and Biotechnology, China Agricultural University, Beijing 100193, China; 2 Institute of Crop Sciences, Chinese Academy of Agricultural Sciences, Beijing 100081, China; 3 Key Laboratory of Molecular Genetics, Guizhou Institute of Tobacco Science, Guiyang 550081, China; 4 Department of Plant Pathology, Kansas State University, Manhattan, KS 66502, USA

**Keywords:** miRNA, siRNA, DNA methylation, non-additive, heterosis, maize, plant height

## Abstract

Heterosis is a complex biological phenomenon regulated by genetic variations and epigenetic changes. However, the roles of small RNAs (sRNAs), an important epigenetic regulatory element, on plant heterosis are still poorly understood. Here, an integrative analysis was performed with sequencing data from multi-omics layers of maize hybrids and their two homologous parental lines to explore the potential underlying mechanisms of sRNAs in plant height (PH) heterosis. sRNAome analysis revealed that 59 (18.61%) microRNAs (miRNAs) and 64,534 (54.00%) 24-nt small interfering RNAs (siRNAs) clusters were non-additively expressed in hybrids. Transcriptome profiles showed that these non-additively expressed miRNAs regulated PH heterosis through activating genes involved in vegetative growth-related pathways while suppressing those related to reproductive and stress response pathways. DNA methylome profiles showed that non-additive methylation events were more likely to be induced by non-additively expressed siRNA clusters. Genes associated with low-parental expression (LPE) siRNAs and trans-chromosomal demethylation (TCdM) events were enriched in developmental processes as well as nutrients and energy metabolism, whereas genes associated with high-parental expression (HPE) siRNAs and trans-chromosomal methylation (TCM) events were gathered in stress response and organelle organization pathways. Our results provide insights into the expression and regulation patterns of sRNAs in hybrids and help to elucidate their potential targeting pathways contributing to PH heterosis.

## 1. Introduction

Heterosis or hybrid vigor is a complex phenomenon in which F_1_ hybrids exhibit superior phenotypic characteristics over their homozygous parental lines, such as growth rates, biomass, grain yield, and stress resistance [1]. Although it has been widely exploited in the breeding of some crop species, including maize, rice, and pigeonpea, over the past decades, the molecular basis underlying the occurrence of heterosis is still unclear. Three major hypotheses, dominance, overdominance, and epistasis, have been proposed at the genetic level [2]. However, these theories were insufficient to explain the molecular basis of heterosis due to a lack of connections to the underlying molecular mechanisms [3]. To date, it is well accepted that heterosis is attributable to gene expression changes, whereas epigenetic modifications (such as DNA methylation, histone modifications, and sRNA regulations) have recently been proposed as a potential factor in the increase of hybrid performance [1,3].

Gene silencing modulated by sRNAs has been found to play critical roles in diverse biological processes in plants. The functions of two major classes of sRNA, microRNAs (miRNAs) and small interfering RNAs (siRNAs), have been well-studied in flowering plants. miRNAs are 21–22-nt endogenous sRNAs produced from single-stranded RNA (ssRNA) stem-loop precursors by *DICER-like 1* (*DCL1*) that act as negative regulators of gene expression at the post-transcription stage by degradation or translational inhibition of messenger RNAs (mRNAs) through nucleotide sequence complementarity [4]. miRNA-mediated gene regulation controls many biological processes, such as plant growth, organ development, and stress resistance. Some miRNAs and their target genes have been identified in various crop species by sRNA and degradome sequencing [5,6]. For example, miR166 regulates the leaf polarity by repressing the *rld1* gene, and miR172 mediates the fate of spikelet meristem by targeting the floral transcription factor *ids1* in maize [7]. In rice, miR396 increases the grain size by targeting the *OsGRF8* gene [8]; and miR165/166 modulates the development of leaf and grain in wheat by regulating the expression of the transcription factor *HB-2* [9]. In addition, miR156 modulates the timing of the juvenile-to-adult transition and leaf development in both maize and rice by regulating the transcription factors *SPLs* [10].

Unlike miRNAs, siRNAs processed from double-stranded RNA (dsRNA) precursors are further classified into multiple subtypes based on their biogenesis pathways, among which the 24- and 21–22-nt siRNAs are the most abundant members [4]. The 24-nt siRNAs participate in the RNA-directed DNA methylation (RdDM) pathway and regulate gene expression at the transcription stage by altering the status of DNA methylation, whereas 21–22-nt siRNAs are mainly involved in post-transcriptional silencing [4,11]. RdDM guided by 24-nt siRNAs participates in several biological processes in flowering plants, such as regulation of flowering time, maintenance of genome stability through transposable element (TE) silencing, as well as responses to abiotic stress and pathogens [11,12].

During the past decade, the relationship between sRNAs and heterosis has been intensively investigated in crops. Non-additively expressed miRNAs play a key role in heterosis via post-transcriptional regulation. For instance, the transgressive expression of miRNAs identified in tomato hybrids is associated with enhanced salt tolerance [13]; non-additively repressed miRNAs and corresponding targets mediate phytohormone response in hybrids of *Brassica napus* [14]; non-additively expressed miRNAs and their targets related to photosynthesis contribute to jointing stage heterosis in maize [7]; and targets of dominantly low-parental expression miRNAs which are enriched in leaf development pathways may account for biomass heterosis in Chinese cabbage [15]. In addition, 24-nt siRNAs induce non-additive DNA methylation in hybrids through allelic interaction in the form of trans-chromosomal methylation (TCM) and demethylation (TCdM) [16,17], which contribute to heterosis in a number of crops. For example, 24-nt siRNA-induced TCM/TCdM events result in enhanced growth and adaptation of maize hybrids [17]. The majority of enhanced expressed 24-nt siRNA clusters in *B. napus* hybrids participated in heterosis through TCM [14]; similar findings in pigeonpea also substantiated the role of TCM and TCdM in heterotic growth [18]. However, it is unclear how sRNAs and their potential targets work synergistically on heterosis because of the lack of sufficient data from multi-omics and our poor understanding of key genes and pathways underlying the traits of interest.

Maize is one of the most excellent model species for studying the molecular basis of heterosis owing to its high level of intraspecific morphological and genetic diversity [19]. Heterosis utilization efficiency in maize can be enhanced by crossing lines from different heterotic groups, usually resulting in superior hybrid performance in traits, such as plant height (PH), biomass, flowing time, and grain yield [20]. So far, several famous heterotic groups have been formed during the modern breeding of commercial hybrid cultivars, such as Stiff Stalk (SS), Non-Stiff stalk (NSS), and Iodent in the United States, and Sipingtou (SPT) in China [21]. As one of the most heritable and easily measured traits in maize, PH is an ideal choice to investigate the molecular mechanism of heterosis. As an important factor of maize plant architecture, an optimal PH could help increase yield by improving the tolerance of high-density planting [22,23]. Therefore, understanding the molecular basis of maize PH heterosis is of great theoretical and practical importance. Several PH-related genes have been identified in maize. For instance, *br2* regulates PH by hindering the polar transportation of auxin [24]; *Dwarf8* and *Dwarf9* that encode DELLA proteins affect PH by repressing the gibberellin (GA) pathway [22,23]; *nana plant1* controls PH by modulating brassinosteroid synthesis [22,23]; the GA-related gene *ZmGA3ox2* reduces PH without influencing yield-associated traits or flowering time [22,23]; *ZmPIN1a* overexpression reduces PH and internode length by regulating auxin spatiotemporal asymmetric distribution [25]; dysfunction of the ethylene biosynthetic gene *ZmACS7* reduces PH and leaf angle by increasing ethylene contents [26]; and *ZmGRF10* affects PH through the regulation of cell proliferation by interacting with GIF proteins [27]. Such studies laid a solid foundation for further exploring the PH heterosis of maize.

In this context, the present study aimed to explore the potential underlying mechanisms of sRNAs in maize PH heterosis by an integrative analysis with sRNAome, transcriptome, and DNA methylome data of tip internodes from two inbred maize lines and their hybrids. We identified a large number of non-additively expressed miRNAs and siRNAs in the hybrid and observed their regulatory effects on putative target genes and genomic regions. We found that genes involved in stress response and reproductive development were more likely to be down-regulated, whereas genes associated with the development process, nutrients, and energy metabolism tended to be up-regulated in the hybrid under different regulatory patterns of miRNAs and siRNAs. Overall, our results provide a better understanding of the important roles of sRNAs in PH heterosis in maize.

## 2. Results

### 2.1. Maize Hybrids Exhibit Significant PH Heterosis

**Figure 1:**
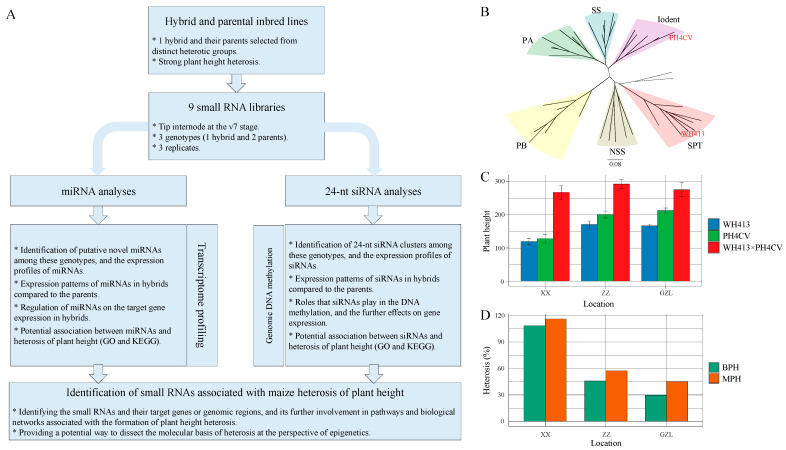
(**A**) Workflow of the present study. (**B**) Phylogenetic tree of maize inbred lines. The selected parental lines, WH413 and PH4CV, are marked in red. (**C**) Comparison of PH between hybrids (WH413×PH4CV) and their parental lines in three environments. (**D**) Distribution of mid-parent heterosis (MPH) and better-parent heterosis (BPH) of plant height (PH) in three environments. In C and D, XX, ZZ, and GZL are the environments of Xinxiang, Henan province, Zhuozhou, Hebei province, and Gongzhuling, Jilin province, respectively.

To identify sRNAs associated with maize PH heterosis, we designed a workflow focusing on miRNA and 24-nt siRNA (Figure 1A). To start with, two inbred lines of different heterotic groups, WH413 from SPT and PH4CV from Iodent, were chosen as the female and male parental lines, respectively, for the heterosis study (Figure 1B). The hybrids exhibit significantly increased PH compared with both parental lines under all three environments in Xinxiang, Zhuozhou, and Gongzhuling (Figure 1C). To evaluate the level of PH heterosis, mid-parent heterosis (MPH) and better-parent heterosis (BPH) were calculated, which ranges from 29.67–108.61% and 45.35–116.29% in the three environments, respectively (Figure 1D). These results demonstrate that the hybrid stably shows significant PH heterosis over parents in different environments.

### 2.2. Identification and Expression Patterns of miRNAs in a Maize Hybrid

**Figure 2:**
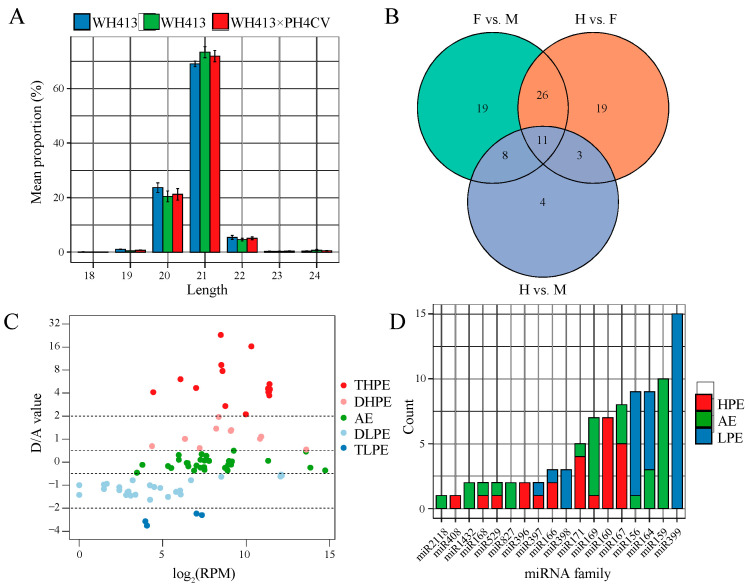
Expression features of miRNAs in maize hybrid relative to its parental lines. (**A**) Distribution of the length of miRNA reads. (**B**) Venn diagram of differentially expressed miRNAs (DEMs) from the three groups of pair-wise comparisons: female vs. male (F vs. M), hybrid vs. female (H vs. F), and hybrid vs. male (H vs. M). (**C**) Scatter plot of DEMs of the five expression patterns: dominant high-parent expression (DHPE), transgressive high-parent expression (THPE), dominant low-parent expression (DLPE), transgressive low-parent expression (TLPE), and additive expression (AE). (**D**) Distribution of members for each miRNA family based on diverse expression patterns of miRNAs, including two high-parent expression (HPE) patterns, two low-parent expression (LPE) patterns, and an additive expression (AE) pattern.

Analyses were performed in both the hybrid and parental lines using the tip internode tissue, which had active gene expression and close association with plant growth, to explore the potential contribution of sRNAs to heterosis for PH, sRNAome, transcriptome, and genomic methylation. A total of 119.25 million raw reads are obtained from nine sRNA libraries for the hybrid and its two parental lines, with an average size of 13.25 million. After removing low-quality reads, adapter sequences and reads mapped to other types of sRNAs (rRNA, scRNA, snoRNA, snRNA, and tRNA), an average of 11.94 million reads with sizes ranging from 18- to 24-nt were selected for identifying the miRNAs. A total of 314 known miRNAs from 30 families and three novel miRNAs are identified in the hybrid and parental lines (Table S1). Among the nine sRNA libraries, an average of 210,407.78 reads are perfectly matched to the identified miRNAs, showing a significant peak at 21-nt, followed by 20-nt and 22-nt (Figure 2A). The number of reads for each miRNA is normalized to reads per million mapped reads (RPM). At the significance level of the adjusted *p*-value (FDR) < 0.05 and fold change > 2, 64, 69, and 26 differentially expressed miRNAs (DEMs) are identified between female vs. male (F vs. M), between hybrid vs. female (H vs. F), and between hybrid vs. male (H vs. M), respectively (Figure 2B).

To identify heterosis-related miRNAs, we classified these DEMs into five groups according to their expression patterns based on the ratio of dominance to additivity, including two HPE patterns (DHPE, dominant high-parent expression; THPE, transgressive high-parent expression), two LPE patterns (DLPE, dominant low-parent expression; TLPE, transgressive low-parent expression), and additive expression (AE) patterns. DEMs from F vs. M group, or commonly up-regulated/down-regulated ones from the H vs. M and H vs. F groups, were considered to distinguish the expression patterns. A total of 90 miRNAs are identified, including 25 HPE miRNAs (10 DHPE and 15 THPE), 34 LPE miRNAs (30 DLPE and 4 TPLE), and 31 AE miRNAs. Interestingly, HPE miRNAs exhibit overall higher expression levels in the hybrid than LPE miRNAs (Figure 2C and Table S2). A total of 18 miRNA families with at least one selected DEM member were chosen for further analysis to characterize the expression pattern of these miRNAs based on their families. As shown in Figure 2D, 14 families contain at least one non-additive expressed member, and most exhibit only one type of non-AE pattern (HPE or LPE), except for the miR397 and miR166 families. These findings suggest that the non-AE patterns of miRNAs are conserved in most miRNA families.

### 2.3. Non-Additive miRNAs Might Contribute to Heterosis by Regulating the Targets of Distinct Pathways

To investigate the biological functions of these heterosis-related miRNAs, we aimed to find their potential target genes. The targets of miRNAs were first screened with degradome sequencing data of pooled hybrid samples. After filtering and analysis of the 15.57 million raw degradome reading, a total of 115 target genes for 140 miRNAs from 22 families are identified, including the *UB2* gene targeted by miR156, *UCE10* targeted by miR399, *AGO1B* targeted by miR168, *CA2P10* targeted by miR169 and *RAR2.7*, and *GL15* targeted by miR172 (Figure S1). Because degradome sequencing cannot capture a sufficient number of cleavage segments generated by some low-expression miRNAs, we could not determine their targets by degradome analysis. psRNATarget was applied to predict the targets of miRNAs with the reference transcriptome to identify the putative missing targets, by which 1170 target genes of 195 miRNAs from 19 families are additionally identified. In total, 1220 targets of 248 miRNAs from 25 families are finally screened out by merging the results from both approaches, with 53 targets of 75 miRNAs shared by both approaches (Table S3).

**Figure 3:**
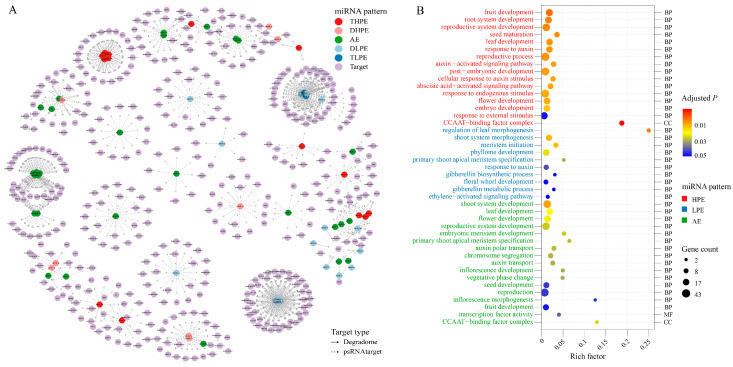
Target genes of miRNAs with distinguishable expression patterns. (**A**) Networks of miRNAs with distinguishable expression pattern and their target genes. The colors of nodes represent the miRNAs with different expression patterns (DHPE, THPE, DLPE, TLPE, and AE), as well as the target genes. Edge types indicate the target genes which were identified by degradome sequencing (solid arrow) or psRNATarget prediction (dashed arrow). (**B**) Enriched GO terms identified with the targets of miRNAs with distinguishable expression patterns. The cut-off of significance is set as an adjusted *p*-value (FDR) < 0.05. The colors of the y-axis labels represent the GO terms significantly enriched by the target genes of miRNAs with different expression patterns (HPE, LPE, and AE). The strings on the right vertical axis (BP, MF, and CC) indicate the GO terms from biological processes, cellular components, and molecular functions, respectively. Circle sizes represent the number of target genes belonging to each GO term. The rich factor is the ratio of the number of miRNA target genes annotated in a GO term to the number of all annotated genes in this term.

Among the 90 miRNAs with distinguishable expression patterns, 75 target genes were identified, which yields 432 genes for further analyses (Figure 3A). Gene Ontology (GO) enrichment analysis showed that the targets of HPE and LPE miRNAs function in quite distinct pathways. The targets of HPE miRNAs are significantly enriched in reproductive growth-related terms (e.g., fruit development, seed maturation, and flower development) and terms of response to endogenous or external stimulus, whereas those of LPE miRNAs focused on vegetative growth-related pathways (e.g., leaf morphogenesis, shoot morphogenesis, and meristem initiation). Additionally, differences in enrichment are found in hormone-related pathways. Target genes of HPE miRNAs are found to act on the abscisic acid-activated signaling pathway, which is associated with the response to abiotic stresses. However, the targets of LPE miRNAs tend to play a role in gibberellin biosynthetic and metabolic processes, which regulate plant developmental processes. In addition, the targets of AE miRNAs are significantly enriched in both reproductive- and vegetative growth-related terms (Figure 3B). Combining the fact that miRNAs play as negative regulators of gene expression through post-transcriptional gene silencing, and the findings obtained above that HPE miRNAs have higher expression levels than LPE miRNAs in hybrid (Figure S2), our results suggest that the non-additive expressed miRNAs might repress the reproductive pathways and stress response pathways while simultaneously activating the vegetative growth-related pathways at the present developmental stage in the hybrid. The specific changes in these pathways might contribute to PH heterosis.

To further analyze the functional modes of miRNA targeting genes in the hybrid, we characterized their expression patterns with transcriptome profiles. A total of 5290 differentially expressed genes (DEGs) were grouped into five expression patterns, adopting the similar classification of miRNA expression patterns described above, including two HPE patterns (TPHE: 345 DEGs; DHPE: 866 DEGs), two LPE patterns (TLPE: 303 DEGs; DLPE: 1107 DEGs), and the AE pattern (2669 DEGs) (Figure S2; Table S4). Among these, 62 DEGs out of the 432 targets of heterosis-related miRNAs could be classified into the above-mentioned expression patterns and were further considered for joint analysis with the expression patterns of miRNAs. We subsequently identified 12 miRNA target pairs in which the miRNAs and their target genes show opposite expression patterns in hybrids (Table 1). Among these pairs, seven genes regulated by five LPE miRNAs exhibit HPE patterns, including genes related to plant growth and nutrient metabolisms, such as *GA20ox2*, *PROT1*, and *PHO2*. Whereas the remaining five LPE genes regulated by three HPE miRNAs contain genes related to pollen development and stress response, such as *ARF17*, *HSP70-6*, and *GB1*. These examples further support the finding that non-additively expressed miRNAs tended to activate the vegetative growth-related pathways while suppressing the reproductive pathways and stress response pathways.

### 2.4. Identification of siRNA Clusters and Their Relationship with DNA Methylation in a Maize Hybrid

**Figure 4:**
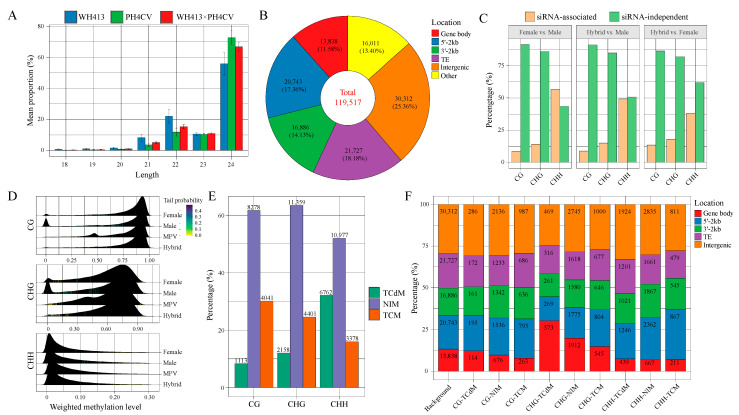
(**A**) Length distribution of siRNA reads. (**B**) Donut plot of 24-nt siRNA clusters based on the types of genomic feature, including gene body, 2 kb of 5′ upstream (5′-2kb), 2 kb of 3′ downstream (3′-2kb), TE, intergenic, and other uncharacterized (other) regions. (**C**) The proportion of siRNA-associated or siRNA-independent differentially methylated regions (DMRs). (**D**) Ridgeline plot of the methylation level of siRNA clusters in the hybrid and the two parental lines, as well as the mid-parental value (MPV). (**E**) Distribution of methylation patterns of siRNA clusters for the three cytosine contexts (CG, CHG, and CHH), including the patterns of trans-chromosomal methylation (TCM), trans-chromosomal demethylation (TCdM), and non-interactive methylation (NIM). (**F**) Distribution of the proportions of siRNA clusters with diverse methylation patterns along different genomic features. The bars represent the sets of siRNA clusters associated with different methylation patterns (TCM, TCdM, and NIM) of the three contexts (CG, CHG, and CHH), as well as the background set of siRNAs (all expressed siRNA clusters). Rectangle colors represent the types of associated genomic features.

To identify the siRNAs involved in the present maize hybrid combination, we merged all the 105.58 million clean reads from the nine sRNA libraries together after removing the miRNA reads, aligned the reads to the maize reference genome, and identified candidate siRNAs using ShortStack. The RPM levels of candidate siRNAs were estimated for each sample. After filtering the candidates with RPM > 2 in at least two separate samples, a total of 124,317 discrete siRNA clusters are selected (Table S5). The clean reads that perfectly matched siRNA clusters in the hybrid and its parents are mostly of the 24-nt types, followed by the 22- and 23-nt types (Figure 4A). The siRNA clusters are defined as 18- to 24-nt clusters according to their dominant reads by ShortStack. Based on ShortStack, the majority of siRNA clusters are classified as 24-nt clusters (119,517, 96.14%), followed by 22-nt clusters (4089, 3.29%) (Figure S3). Most siRNA clusters (86.65%) are shorter than 1 kb (Figure S4). We then surveyed the locations of 24- and 22-nt siRNA clusters relative to protein-coding genes. A large proportion of 24-nt siRNA clusters (43.07%) are enriched in protein-coding genes and their flanking regions, including 17.36% within 5′ upstream 2 kb regions, 11.58% in the gene body and 14.13% in 3′ downstream 2 kb regions (Figure 4B), which indicates a close relationship between 24-nt siRNA clusters and protein-coding genes in the maize genome. Meanwhile, 18.18% and 25.36% of 24-nt siRNA clusters co-localize with transposable elements (TE) and intergenic regions, respectively (Figure 4B). However, most 22-nt siRNA clusters (53.14%) are enriched in TE regions (Figure S5), suggesting the distinct biological functions of the two types of siRNA clusters.

As 24-nt siRNAs play a central role in RdDM, we then investigated the DNA methylation changes associated with 24-nt siRNA clusters using whole-genome bisulfite sequencing of the hybrid and its parental lines. A location-based comparison between 24-nt siRNA clusters and genome-wide differentially methylation regions (DMRs) reveals a significantly higher proportion of siRNA cluster-associated DMRs in the CHH context than in the CG and CHG contexts. Such divergence was observed in the DMRs identified from the F vs. M, H vs. M, and H. vs. F groups, indicating that 24-nt siRNA clusters may play a key role in the methylation of the CHH context (Figure 4C). By comparing the methylation levels of siRNA clusters between the hybrid and its parents, we found that both CG and CHG weighted methylation levels (WML) of siRNA clusters in the hybrid are significantly higher than the mid-parental values (MPVs) of parental WML (Wilcoxon rank sum test, *p* < 2.2 × 10^−16^ for both CG and CHG), whereas the CHH WML of siRNA clusters in the hybrid is significantly lower than MPVs (Wilcoxon rank sum test, *p* < 2.2 × 10^−16^) (Figure 4D). To further explore non-additive methylation in the siRNA clusters, which might be associated with PH heterosis, we classified the methylation patterns of siRNA clusters in the hybrid into three patterns (TCM, TCdM, and NIM) based on methylation differences between the hybrid and its parental lines (see the Materials and Methods). The results showed that the proportions of TCM are significantly higher than that of TCdM for both CG and CHG but changed to the opposite for CHH (Figure 4E; Table S6). These results suggest that the CG and CHG contexts of siRNA clusters are more likely to be hyper-methylated in the hybrid by TCM events, whereas CHH contexts tend to be hypo-methylated through TCdM events. In addition, we also determined the distribution of siRNA clusters with assigned methylation patterns along genomic features. Compared with the whole genome background, siRNA clusters associated with CG-TCM and CHH-TCM events are significantly enriched in 5′ upstream regions (23.57% and 29.76%, respectively, compared with 20.04% of the background), whereas siRNA clusters associated with CHG-TCdM events show remarkable enrichment in gene body regions (30.35% compared with 13.37% of the background) (Figure 4F; Table S6).

### 2.5. Non-Additively Expressed siRNA Might Contribute to PH Heterosis by Modulating the Methylation of Co-Localized Genes

Principal component analysis (PCA) of siRNA expression revealed a clear separation of the hybrid and its parental lines, providing us with a solid basis to investigate the role of siRNAs in PH heterosis (Figure S6). To find the potential heterosis-related siRNA clusters, a similar approach as adopted in the miRNA section was performed. As a result, 64,534 differential expressed siRNA clusters (DESs) from the F vs. M group or commonly up/down-regulated between the H vs. M and H vs. F groups are identified and further classified into five expression patterns based on the ratio of dominance to additivity. In total, 10,786 HPE siRNA clusters (9366 DHPE and 1420 THPE), 9238 LPE miRNAs (8936 DLPE and 302 TPLE), as well as 44510 AE miRNAs are identified. Similarly to miRNAs, the HPE siRNA clusters show higher expression levels in the hybrid than the LPE siRNA clusters (Figure S7 and Table S6).

**Figure 5:**
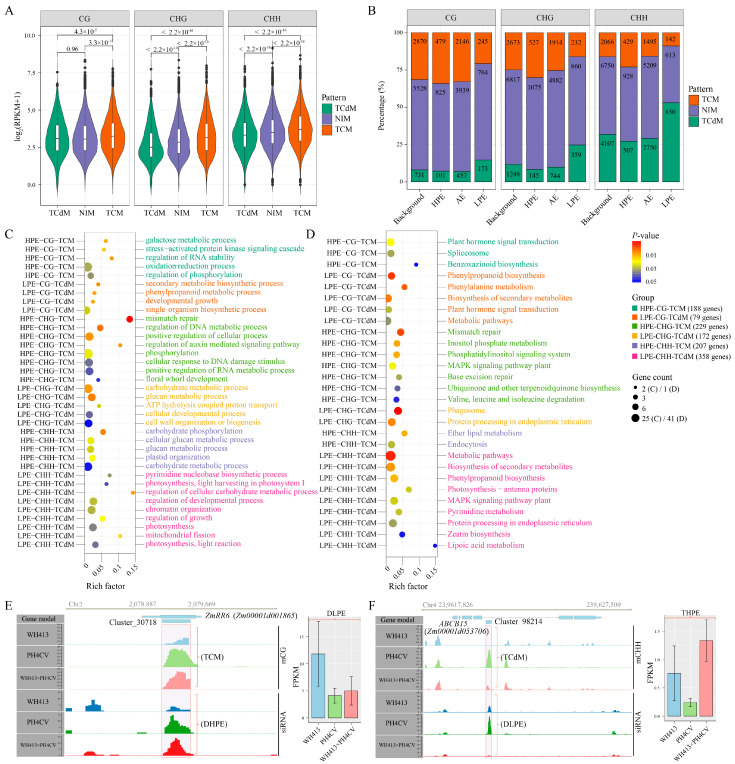
(**A**) Comparisons of siRNA expression levels among methylation patterns (TCM, TCdM, and NIM) for the three cytosine contexts (CG, CHG, and CHH). The *p*-value of the Student’s *t*-test for each comparison is shown in the plot. (**B**) Distribution of the proportions of siRNA clusters grouped by their expression patterns (HPE, LPE, and AE) and methylation patterns (TCM, TCdM, and NIM) for the three cytosine contexts (CG, CHG, and CHH). The bars in each panel represent the sets of siRNA clusters with different expression patterns, as well as the background set of siRNAs (all expressed siRNA clusters) for a given cytosine context. The colors of rectangles represent the methylation patterns. (**C**,**D**) Enriched GO terms and KEGG pathways identified with the protein-coding genes associated with non-additively expressed and methylated siRNA clusters, respectively. A relaxed cut-off of significance was set as *p*-value < 0.05. Strings on the y-axis indicate the siRNA clusters grouped by their expression and methylation patterns in the three cytosine contexts. The colors of the right vertical axis labels represent the GO terms (**C**) or KEGG pathways (**D**) significantly enriched by diverse sets of genes associated with the siRNA clusters grouped by their expression and methylation patterns of cytosine contexts. The colors of dots represent the significance levels of enrichment analyses. (**E**,**F**) are the relationship between the expression patterns of siRNA clusters and their methylation patterns (left panel) and the effects on the expression of associated protein-coding genes (right panel) for siRNA clusters, Cluster_30718 and Cluster_98214, respectively. The blue, green, and red histograms represent the methylation levels of siRNA clusters (light colors, top three histograms on the left panel), the expression levels of siRNA clusters (dark colors, bottom three histograms on the left panel), or the expression levels of siRNA-associated genes (light colors, three bars on the right panel) in the two parental lines (WH413 and PH4CV) and the hybrid (WH413×PH4CV).

To explore whether the siRNA clusters contributed to PH heterosis by affecting genomic methylation, we aimed to determine the relationship between the expression of siRNA clusters and their methylation features. As shown in Figure 5A, the TCM siRNA clusters have significantly higher expression levels than those with the other two methylation patterns (NIM and TCdM) in the hybrid for all three cytosine contexts. Meanwhile, the TCdM siRNA clusters show significantly lower expression levels than those of the other two methylation patterns for the CHG and CHH, but not the CG, contexts. These results imply that higher expression of the siRNA clusters is related to hyper-methylation of the co-localized regions. We then grouped the siRNA clusters by their expression patterns (HPE, LPE, and AE) and methylation patterns (TCM, TCdM, and NIM) for the three cytosine contexts (CG, CHG, and CHH) and further examined the potential association between non-additively expressed siRNA clusters and their methylation patterns. Compared with the background, the LPE siRNA clusters are significantly aggregated in the TCdM pattern for all three contexts (*p* = 2.1 × 10^−15^ for CG; *p* < 2.2 × 10^−16^ for CHG; *p* < 2.2 × 10^−16^ for CHH) (Figure 5B). In contrast, HPE siRNA clusters are significantly enriched in the TCM pattern for CHG and CHH (*p* = 0.13 for CG; *p* = 3.4 × 10^−5^ for CHG; *p* = 8.4 × 10^−14^ for CHH) (Figure 5B). The results suggest that the non-additively expressed siRNA clusters played an important role in non-additive methylation patterns.

We then speculated that such non-additively expressed and methylated siRNA clusters might contribute to PH heterosis. To dissect the potential functions of these siRNA clusters, we focused on the 963 protein-coding gene-associated siRNA clusters belonging to the following six groups: three groups in which the siRNA clusters showed HPE and TCM (HPE-CG-TCM, HPE-CHG-TCM, and HPE-CHH-TCM), and three groups in which the siRNA clusters showed LPE and TCdM (LPE-CG-TCdM, LPE-CHG-TCdM, and LPE-CHH-TCdM) (Table S7). A total of 940 protein-coding genes associated with these 963 eligible siRNA clusters (including siRNA clusters co-located with gene body, the 5′-2kb and 3′-2kb) are identified and then subject to GO and KEGG enrichment analyses. Both analyses reveal that the genes associated with the three HPE and TCM groups of siRNA clusters are more likely to be enriched in terms related to stress response, response to cellular stimulus, and organelle organization (Figure 5C,D). However, genes associated with the three LPE and TCdM groups of siRNA clusters tend to function in the secondary metabolite process, developmental process, carbohydrate metabolism, and energy metabolism (Figure 5C,D). These results showed that the HPE siRNA clusters repressed the genes related to stress response by siRNA-mediated hyper-methylation, whereas the LPE siRNA clusters activated the genes related to development, photosynthesis, and metabolism through siRNA-mediated hypo-methylation in the hybrids. This finding suggests that siRNA clusters may contribute to PH heterosis through complex and extensive regulations on their associated genes and relevant biological processes.

Because 24-nt siRNAs regulated gene expression at the transcription stage by altering the DNA methylation status, we subsequently surveyed the non-additively expressed genes that were associated with non-additively expressed and methylated siRNA clusters. As a result, 75 eligible genes are identified from the pools associated with the six groups of siRNA clusters (Table S7). Even though the relatively low counts of genes reduced the statistical power to detect enrichment, we still found that LPE genes are enriched in the group of HPE-CG-TCM (Fisher’s exact test, *p* = 0.029), and HPE genes are enriched in the group of LPE-CHH-TCdM (Fisher’s exact test, *p* = 0.036) (Figure S8). These results suggest that LPE siRNA clusters are more likely to activate their associated genes through non-additively hypor-methylation in the CG context, and HPE siRNA clusters might repress their associated genes through non-additively hyper-methylation in the CHH context. Among these siRNA-gene pairs, we summarize several genes whose expression patterns could be explained by a combination of siRNA expression patterns and methylation patterns, which might contribute to PH heterosis (Table 2, Figure 5E,F). For instance, genes related to biological processes, such as stress response (*GSTU25*, *TIFY10B*), disease resistance (*GBF3*), and reproductive development (*ZmRR6*, *KNR6*), are repressed in hybrids by siRNA-associated TCM events. Meanwhile, genes related to plant growth (*IBL1*) and metabolism (*G6PD2*, Zm00001d040061/Pectinacetylesterase) were activated by siRNA-associated TCdM events.

## 3. Discussion

Heterosis is a complex trait affected by multiple layers of biological processes, and traditional genetic or genomic analysis alone is insufficient to dissect the molecular basis. Recently, studies have focused on the effect of epigenetic regulation on plant heterosis, including small RNAs-mediated gene silencing by miRNAs [7,14,15,18,28] and siRNAs [14,17,18]. In the present study, an integrative and comprehensive analysis was carried out focusing on the regulatory mechanism of sRNAs and their potential targets in PH heterosis. The results showed that non-additively expressed miRNAs activated vegetative growth-related pathways but suppressed the reproductive and stress response pathways in the hybrid. Meanwhile, genes associated with LPE siRNAs and TCdM events tended to accumulate in the developmental processes, whereas genes associated with HPE siRNAs and TCM events were enriched in stress response and organelle organization pathways. Our findings demonstrated that both miRNAs and siRNAs together contribute to PH heterosis in maize.

### 3.1. PH Serves as an Excellent Model Trait for Studying Maize Heterosis

Maize exhibits remarkable heterosis for a number of traits, among which PH stands out as one of the most heritable and easily measured. In the present study, we derived F_1_ hybrids by a single-cross of WH413 and PH4CV, which were selected from two distinct heterotic groups following the heterosis pattern SPT × Iodent. The hybrids showed distinguished heterosis and stable heredity for PH in three environments of north China (Figure 1D), making the parent-hybrid combination an ideal research target for heterosis studies.

Biologically, maize PH is usually determined by two parameters, average internode number and internode length. Internode number is driven by the differentiation of shoot meristem, and internode length is attributed to intercalary cell expansion [29]. In the past few decades, a number of genes from diverse pathways that modulate internode elongation have been identified in maize and other plant species, such as the maize auxin pathway-related genes *vt2*, *br2*, *brevis plant1* (*bv1*), and *ZmPIN1a* [25]; maize GA-associated genes *anther ear1* (*an1*), *dwarf1* (*d1*), *d3*, *d5*, *D8, D9* and *ZmGA3ox2* [22,23]; BR-related gene *Dwarf* (*D*) in tomato, *dwarf1* (*brd1*) in rice, and *nana plant1* in maize [23,30]; ethylene-related genes *Semidwarf3* (*ZmACS7*) and *zmbri1* in maize, *Uzu1* in barley [26,30]; and cell growth and differentiation-related genes *ZmGRF10*, *gif1*, *ZmTE1*, and *blh12/14* in maize [27,29,31]. The understanding of the key genes and pathways underlying PH makes it an excellent model trait to study the molecular basis of maize heterosis. Therefore, in the present study, the sRNAome, transcriptome, and DNA methylome of tip internodes at the V7 stage were combined for the investigation of PH heterosis in maize.

### 3.2. miRNAs Are Critical Regulators of Maize PH Heterosis

miRNAs involved in plant growth and development are more likely to be differentially regulated in hybrids and show non-additively expression relative to their parental lines, which may be related to heterosis [3]. In this study, 59 non-additively expressed miRNAs (25 HPE and 34 LPE miRNAs) were identified by comparing the hybrid to the parental lines based on D/A ratios. The target genes of miRNAs were identified with both degradome and prediction-based approaches. GO enrichment analysis showed that targets involved in vegetative growth-related pathways were activated by LPE miRNAs, whereas targets related to reproductive pathways and stress response pathways were repressed by HPE miRNAs simultaneously. Such difference between the two target sets suggested the important roles of non-additively expressed miRNAs in heterosis by regulating distinct physiological processes.

Recently, several studies in *Arabidopsis* [32], wheat [33], and rapeseed [14] showed that non-additively expressed miRNAs in hybrids result in non-additively opposing expression of their target genes, which might contribute to the heterosis in various traits. In this study, 12 miRNA-target pairs in which the miRNAs and their target genes exhibited non-additively opposing expression patterns were identified with the transcriptome profiles (Table 1). Among them, seven genes regulated by five LPE miRNAs showed HPE patterns, which might participate in PH heterosis by promoting plant growth. For instance, a conserved miRNA family in the plant kingdom, miR164, was repressed in hybrids, whereas its targets *GA20ox2* and *PROT1* were up-regulated. *GA20ox2*, as a gibberellin biosynthetic gene, promotes plant growth and development [34], and *PROT1* is uniformly expressed in phloem cells and is related to xylem development [35]. This finding is consistent with previous reports that miR164 affects plant growth [7,28]. miR399 participates in phosphate homeostasis and transportation by regulating its target *PHOSPHATE2* (*PHO2*), which plays pivotal roles in phosphorus uptake and plant growth [36]. Loss-of-function of the predominant *PHO2* homolog *TaPHO2-D1* reduces PH in wheat; over-expressed miR399b in maize leads to excessive amounts of P in the shoots and Pi-toxicity [36]. In our study, miR399 was repressed (15 LPE members), and *PHO2* was activated in hybrids, which may improve the transportation and utilization of phosphate, thus increasing PH. Additionally, miR156 controls plant architecture by the miR156/SPL module in grass species and maize, that repressed miR156 enhances internode elongation by inducing *SPLs* expression [10,28]. Consistently, our findings demonstrate that suppressed miR156 resulted in the up-regulation of its target *UB2* in hybrid, which was an *SPL* member and has been reported to participate in phase transition [10,37], leading to faster developmental progress in hybrids than in parental inbred lines.

On the other hand, we also found three HPE miRNAs and five corresponding LPE genes that might affect PH in hybrids by inactivating reproduction and stress response processes. For example, miR160 of the auxin signaling pathway represses the expression of *ARF17*, which might influence pollen development [38]. Genes such as *HSP70-6* and *GB1*, which function in abiotic stress tolerance and disease resistance [39,40], were down-regulated by the activated miR167 in hybrids. To summarize, the functional analysis of the 12 miRNA-target pairs supports our GO enrichment analysis results that genes involved in reproductive and stress response pathways were suppressed, whereas those associated with plant growth and development were activated by miRNAs in hybrids. This phenomenon probably implies less energy allocated to reproduction and stress tolerance; thus, more energy would be available for plant growth, which accounts for PH heterosis in our study. It should be noted that the opposing expression patterns between miRNAs and their targets suggest a regulatory mode through post-transcriptional degradation. Gene translation inhibited by miRNAs is also important, but it was not evaluated in the current study.

### 3.3. The 24-nt siRNAs Play an Important Role in Heterosis by Altering the DNA Methylation Status of Associated Genes

As important regulators affecting DNA methylation through RdDM, 24-nt siRNAs are involved in the alteration of the DNA methylation status of hybrids in plant species, such as *Arabidopsis* [41] and rapeseed [14]. Because siRNAs generated from the methylated parental allele act as trans-regulators to initiate RdDM and result in methylation of the previously unmethylated or hypo-methylated parental allele, they cause non-additive changes in DNA methylation levels [16]. Such non-additive methylation in hybrids is attributed to two mechanisms of allelic interaction, TCM induced by the increased 24-nt siRNA levels and TCdM induced by repressed 24-nt siRNAs [16,42]. In this study, we substantiated the importance of siRNAs in the induction of non-additive methylation events based on two lines of evidence. First, we found that the expression levels of siRNA clusters associated with TCM events for all three sequence contexts were significantly higher than those of siRNA clusters associated with TCdM events (Figure 5A). Second, the LPE and HPE siRNA clusters were significantly associated with the TCdM and TCM patterns, respectively (Figure 5B).

Because DNA methylation plays a prominent role in silencing gene expression at the transcription stage, the alterations in methylation status induced by siRNAs were previously found to be correlated with the expression of associated genes in hybrids. For instance, genes involved in circadian rhythm and plant growth are hypo-methylated and up-regulated in pigeonpea hybrids, whereas genes participating in defense and stress response are hyper-methylated and down-regulated [18]. Stress-responsive genes are repressed by hyper-methylation in maize hybrids [17]. In our study, functional enrichment analyses showed that genes associated with HPE siRNAs and TCM events were enriched in stress response and organelle organization, whereas genes associated with LPE siRNAs and TCdM events gathered in the developmental process, carbohydrate metabolism, and photosynthesis. For example, *GSTU25* is involved in environmental stress response [43], *TIFY10B* responds to alkaline stress [44], *GBF3* participates in disease resistance [45], *ZmRR6* is involved in the development of root and panicles [46], *KNR6* in development of maize ears [47], and all were repressed by HPE siRNAs and TCM events. In contrast, *IBL1* is associated with cell elongation in *Arabidopsis* [48], *G6PD2* is involved in the pentose phosphate pathway [49], and both were activated by LPE siRNAs and TCdM events. Such results are in line with previous studies, revealing the important roles of non-additive methylation induced by siRNAs in heterosis. Nevertheless, it should be noted that the effect of CHH methylation was quite non-canonical in our study. Genes such as *PDK* (involved in energy metabolism) were activated by HPE siRNAs and CHH-TCM events [50], whereas the two stress-responsive genes, *TSIP1* and *PAL*, were repressed by LPE siRNAs and CHH-TCdM events [51,52]. Consistent with our findings, CHH methylation levels also exhibited a weak positive correlation with gene expression in a recent study on eight grass species [53]. Altogether, these results suggest the complex effects of CHH methylation on gene expression.

## 4. Materials and Methods

### 4.1. Plant Materials and Phenotyping

A maize F_1_ hybrid was produced by a single cross between two inbred lines, WH413 and PH4CV, which was in accordance with a heterosis pattern SPT × Iodent. In the summer of 2018, the hybrid and its two parental lines were sown in three environments of north China with two replications, including Gongzhuling (GZL, Jilin province), Zhuozhou (ZZ, Hebei province) and Xinxiang (XX, Henan province). Ten plants in each replicate were evaluated for PH after the flowering stage. The MPH values were calculated following the equation MPH = (F_1_ − MPV)/MPV, where F_1_ is the PH of the hybrid, and MPV is the mean value of the two parental lines. The BPH values were calculated with the equation BPH = (F_1_ − BPV)/BPV, where F_1_ is the PH of the hybrid, and BPV is the PH of the better-parent line.

### 4.2. Construction and Sequencing of sRNA-seq, mRNA-seq, and Degradome Libraries

For each genotype, both the sRNA-seq and mRNA-seq libraries were constructed with three biological replications. In each biological replicate, equal amounts of tissue from the tip internodes with decapitated inflorescence meristem from three randomly selected plants were harvested when the plants grew to the V7 stage. The total RNA of each sample was extracted using the TRIzol reagent (Invitrogen, Waltham, MA, USA). The sRNA and mRNA libraries were constructed using TrueSeq Small RNA Sample Prep Kits and TrueSeq mRNA Sample Prep Kits, respectively, both of which were provided by Illumina (Illumina, San Diego, CA, USA). Single-end sequencing with a read length of 50 bp and paired-end sequencing with a read length of 150 bp was carried out on Illumina HiSeq 2500 sequencer (Illumina, San Diego, CA, USA) for sRNA and mRNA libraries, respectively.

For degradome library construction, we equally mixed total RNAs from 3 hybrid samples to construct a single degradome library, as previously described [54]. In brief, poly (A) RNA was purified from the total RNA using poly-oligo(dT)-attached magnetic beads and reverse-transcribed to first-strand cDNA (New England Biolabs, Ipswich, MA, USA). The cDNA library was sequenced as single-end libraries with a read length of 50 bp for degradome sequencing.

### 4.3. Identification of miRNAs and siRNAs

The raw reads of sRNA libraries were processed to remove low-quality reads and adapter sequences using Cutadapt [55]. Subsequently, the reads matching rRNA, scRNA, snoRNA, snRNA, and tRNA were filtered using bowtie [56]. The reference sequences of 374 maize non-redundant known RNAs were collected by combining the annotations of miRBase (v22.0, 12 March 2018) (https://www.mirbase.org/) and Gramene (Maize genomic GFF of Release 58, 11 August 2018) (https://www.gramene.org/). The clean reads with lengths ranging from 18 to 24-nt were firstly aligned to the reference of known miRNAs by bowtie, allowing only a perfect match to identify known miRNAs. The remaining reads were aligned to a maize reference genome (V4) by bowtie allowing only perfect match to identify novel miRNAs. miRDeep-P2 [57] was performed to identify the novel miRNAs, and only the candidates meeting the strict criteria proposed by the literature [58] were considered novel miRNAs. The RPM values of miRNAs in each sample were estimated with the mapped miRNA reads.

To identify the siRNA clusters, clean reads of the 9 sRNA libraries were merged together after excluding the miRNA reads. The siRNA clusters were identified with the following steps using ShortStack [59]. In brief, the reads were firstly aligned to the maize genome under the unique-weighting mode with no mismatch, which could determine the proper placement of multiple mapped reads based on the frequencies of unique mapping reads within the adjacent regions. Candidate siRNA clusters were de novo identified with default settings. Subsequently, the filtered reads of each sample were separately aligned to the genome to calculate the RPM of candidate siRNA clusters. Only the candidates with RPM > 2 in at least two separate samples were treated as true siRNA clusters.

### 4.4. Differential Expressions and Expression Patterns of miRNA and siRNA

The analyses of differential expression for miRNAs and siRNA clusters were both conducted with DESeq2 [60] under negative binomial generalized linear models. The differentially expressed miRNAs (DEMs) and siRNAs (DESs) were identified with criteria of the adjusted *p*-value (FDR) < 0.05 and fold change > 2 in three groups of pair-wise comparisons (female vs. male, hybrid vs. male, and hybrid vs. female). Only the differentially expressed sRNAs identified from the F vs. M group or the commonly up-regulated or down-regulated ones of the H vs. M and H vs. F groups were considered as candidates which might be closely associated with heterosis. To identify the expression patterns of sRNAs in hybrid, the ratio of dominance to additivity (D/A) for each sRNA in this set of candidates was estimated based on RPM, in which the values of dominance were calculated as RPM_Hybrid_ − RPM_MPV_ (RPM_MPV_ represents the mid-parent value of expression), and the values of additivity were calculated as RPM_HP_ − RPM_MPV_ (RPM_HP_ represents high parent expression). The expression of sRNAs was classified into 5 expression patterns based on the D/A values: (1) THPE, transgressive high-parent expression, with D/A > 2; (2) DHPE, dominant high-parent expression, with D/A < 2 and > 0.5; (3) AE, additive expression, with D/A < 0.5 and > −0.5; (4) DLPE, dominant low-parent expression, with D/A < −0.5 and > −2; and (5) TLPE, transgressive low-parent expression, with D/A < −2.

### 4.5. Identification of miRNA Target Genes

The target genes of miRNAs were first identified with the data of degradome sequencing. The raw reads of the degradome library were processed with Cutadapt by removing the low-quality reads, adapter sequences, and reads mapped to known small RNAs. The clean reads were aligned to the maize reference transcriptome, and the potentially cleaved targets were identified by the CleaveLand 4.0 [61] with a cut-off of *p*-value < 0.05. The cleaved events caused by miRNAs were classified into 4 categories based on the abundance of cleaved reads across the target transcripts. The potential targets of miRNAs were also predicted by psRNATarget [62] against maize reference transcriptome.

### 4.6. Analysis of mRNA-seq Data

The raw reads of mRNA libraries were filtered with Trimmomatic [63] by removing low-quality reads and adapter sequences. Next, the clean reads were aligned to the maize reference genome (V4) by STAR with a 2-pass mode [64]. Only the uniquely mapped reads in each sample were utilized to estimate the gene expressions. Gene expression levels were quantified and normalized as fragments per kb per million reads (FPKM) by StringTie [65]. After filtering out genes with FPKM > 0.5 in at least two samples, the differentially expressed genes (DEGs) were identified by DESeq2 with criteria of the adjusted *p*-value (FDR) < 0.05 and fold change > 2. We adopted a similar approach for sRNAs, the DEGs from the F vs. M group, or the common up-regulated or down-regulated ones of H vs. M and H vs. F groups, and were classified into five expression patterns (THPE, DHPE, AE, DLPE, and TLPE) based on the ratio of dominance to additivity (D/A). GO enrichment analysis was performed using the agriGO v2 online tool [66]. KEGG pathway enrichment analysis was conducted with KOBAS v3.0 [67].

### 4.7. Construction of Bisulfite-seq Libraries and Analysis of Whole-Genome Bisulfite Sequencing (WGBS)-seq Data

For each genotype, the Bisulfite-seq (BS-seq) libraries were constructed with 2 biological replications. The total genomic DNA was extracted using a DNeasy plant mini kit (QIAGEN, Hilden, Germany). Bisulfite conversion of DNA, construction of Bisulfite-seq library, and sequencing were performed at Annoroad Gene Technology (Annoroad, Beijing, China). The clean reads of BS-seq were obtained by Trimmomatic after removing low-quality reads and adapter sequences. Then, the clean reads were aligned to a maize reference genome (V4) by Bismark [68] with parameters of -N 1 -L 30 --score_min L,0,-0.6 -X 1000. Bisulphite conversion of the libraries was evaluated by using the lambda genome as a control. The conversion rates of all samples were greater than 99%, ranging from 99.41% to 99.59%, which suggested the high quality of the BS-seq libraries. The uniquely mapped reads were retained for further analysis. After removing the cloned reads generated by PCR amplification, only the C sites covered with at least 5 reads were considered to estimate the methylation level. The DMRs were identified by metilene [69] under the parameters of -f 2 -c 2 for genome-wide 100 bp non-overlapped bins. The bins contained at least four cytosines and with an adjusted *p*-value (FDR) < 0.05 were regarded as DMRs.

The methylation profiles of siRNA clusters were estimated as the weighted methylation level [70]. The methylation patterns of siRNA clusters in hybrids were detected with the following steps. Firstly, the differentially methylated siRNA clusters were identified with metilene for three groups of pair-wise comparisons (F vs. M, H vs. M, and H vs. F). The differentially methylated siRNA clusters were selected as those containing at least four cytosines and with adjusted *p*-value (FDR) < 0.05 and absolute methylation difference of 0.1, 0.1, and 0.05 for CG, CHG, and CHH contexts, respectively. Secondly, only the differentially methylated siRNA clusters from the F vs. M group, or the common hyper-methylated or hypo-methylated ones of H vs. M and H vs. F groups were subjected to further classification into three methylation patterns following a similar approach adopted by a previous study [16]. In brief, the methylation-based ratios of dominance to additive were calculated with the formula: Met_d/a_ = (WML_Hybrid_ − WML_MPV_)/max(abs(WML_F_ − WML_MPV_, WML_M_ − WML_MPV_)), where WML_Hybrid_, WML_F_, WML_M_, and WML_MPV_ represented the WMLs of hybrid, female, male, and the mid-parent value, respectively. The patterns of siRNA clusters with Met_d/a_ > 0.5 or Met_d/a_ < −0.5 were defined as Trans-chromosomal methylation (TCM) and Trans-chromosomal demethylation (TCdM), respectively, whereas that with Met_d/a_ < 0.5 and > −0.5 was defined as non-interactive methylation (NIM).

## 5. Conclusions

In this study, we investigated the differences in sRNAs levels and their putative target genes between F_1_ hybrids and their parental lines using multi-omics data and performed an integrative analysis on the epigenetic regulation of sRNAs contributing to maize PH heterosis. We found that genes involved in stress response and reproductive development were repressed by HPE miRNAs and TCM events induced by HPE siRNAs, but genes involved in the development process, nutrients, and energy metabolism were activated by LPE miRNAs and TCdM events induced by LPE siRNAs. Our work not only provides a new insight into the regulatory pattern of sRNAs on heterosis but also sheds light on the key small RNA-associated heterotic genes and pathways for maize PH.

## Figures and Tables

**Table 1:** The non-additive alteration of genes targeted by non-additively expressed miRNAs.

Family	Representative miRNAs	Express Pattern of miRNA ^a^	D/A Ratio of miRNA ^b^	Target Gene ID	Gene Name	Express Pattern of Target ^c^	D/A Ratio of Target ^d^
miR164	zma-miR164f-5p	DLPE	−0.79	Zm00001d002101	*GA20ox2*	DHPE	0.92
				Zm00001d042438	*PROT1*	DHPE	1.33
		DLPE	−0.79	Zm00001d047973	*CYP18-1*	DHPE	0.77
miR399	zma-miR399c-3p	DLPE	−1.37	Zm00001d038972	*PHO2*	THPE	19.8
	zma-miR399i-5p	DLPE	−1	Zm00001d003477	*CRY2*	THPE	10.59
miR156	zma-miR156k-5p	DLPE	−0.64	Zm00001d022275	*NPSN13*	DHPE	1.19
	zma-miR156h-5p	TLPE	−3.39	Zm00001d031451	*UB2*	THPE	8.51
miR160	zma-miR160g-5p	THPE	5.23	Zm00001d002929	*ARF17*	TLPE	−15.86
				Zm00001d025225	*-*	DLPE	−1.25
miR167	zma-miR167d-3p	THPE	4.66	Zm00001d028630	*HSP70-6*	DLPE	−0.95
	zma-miR167c-3p	THPE	16.29	Zm00001d029408	*GB1*	DLPE	−1.81
				Zm00001d040731	*GDSL-like lipase*	TLPE	−2.1

^a^ and ^c^ indicate the expression patterns of miRNAs and their target genes, respectively. The expression patterns include dominant high-parent expression (DHPE), transgressive high-parent expression (THPE), dominant low-parent expression (DLPE), transgressive low-parent expression (TLPE), and additive expression (AE). ^b^ and ^d^ indicate the D/A ratios of miRNAs and their target genes, respectively, which are estimated as the ratio of dominance to additivity (D/A).

**Table 2:** Selected list of non-additively expressed genes associated with non-additively expressed and methylated siRNA clusters.

Group ^a^	siRNA ID	Express Pattern of siRNA Cluster	D/A Ratio of siRNA Cluster	Cytosine Context	Methylation Pattern	D/A Ratio of Methylation Level	Feature ^b^	Gene ID	Gene Name	Expression Pattern of Gene	D/A Ratio of Gene
HPE-CG-TCM	Cluster_30718	HPE	0.85	CG	TCM	0.55	Gene body	Zm00001d001865	*ZmRR6*	DLPE	−0.77
HPE-CG-TCM	Cluster_137039	HPE	0.62	CG	TCM	0.65	5’-2kb	Zm00001d039065	*GBF3*	DLPE	−0.71
HPE-CG-TCM	Cluster_28403	HPE	0.72	CG	TCM	0.8	3’-2kb	Zm00001d034356	*GSTU25*	DLPE	−1.94
HPE-CG-TCM	Cluster_80610	HPE	1.07	CG	TCM	1.02	3’-2kb	Zm00001d049630	*Galactose oxidase*	TLPE	−4.55
HPE-CG-TCM	Cluster_127623	HPE	1.11	CG	TCM	0.92	5’-2kb	Zm00001d036602	*KNR6*	DLPE	−0.87
HPE-CHG-TCM	Cluster_28403	HPE	0.72	CHG	TCM	0.68	3’-2kb	Zm00001d034356	*GSTU25*	DLPE	−1.94
HPE-CHG-TCM	Cluster_153805	HPE	0.59	CHG	TCM	0.68	3’-2kb	Zm00001d022530	*EIN3*	DLPE	−1.07
HPE-CHH-TCM	Cluster_8216	HPE	0.63	CHH	TCM	1.06	5’-2kb	Zm00001d029448	*TIFY10B*	DLPE	−0.98
HPE-CHH-TCM	Cluster_186180	HPE	0.78	CHH	TCM	1.24	3’-2kb	Zm00001d048145	*PDK*	DHPE	1.03
LPE-CG-TCdM	Cluster_191145	LPE	−0.95	CG	TCdM	−0.55	5’-2kb	Zm00001d024166	*IBL1*	THPE	11.05
LPE-CG-TCdM	Cluster_56715	LPE	−0.74	CG	TCdM	−0.53	Gene body	Zm00001d040061	*Pectinacetylesterase*	DHPE	0.96
LPE-CHH-TCdM	Cluster_98214	LPE	−0.92	CHH	TCdM	−0.9	Gene body	Zm00001d053706	*ABCB15*	THPE	3.24
LPE-CHH-TCdM	Cluster_8459	LPE	−0.95	CHH	TCdM	−1	Gene body	Zm00001d029502	*G6PD2*	THPE	16.39
LPE-CHH-TCdM	Cluster_146598	LPE	−0.62	CHH	TCdM	−0.51	Gene body	Zm00001d020538	*TSIP1*	DLPE	−0.64
LPE-CHH-TCdM	Cluster_87940	LPE	−0.96	CHH	TCdM	−1	Gene body	Zm00001d051161	*PAL*	TLPE	−2.9

^a^ indicate the siRNA cluster belonging to one of the groups by siRNA expression patterns (HPE and LPE) and methylation patterns (TCM and TCdM) for the three cytosine contexts (CG, CHG, and CHH). ^b^ indicate the genomic features associated with the siRNA clusters, including gene body, 2 kb of 5′ upstream (5′-2kb), and 2 kb of 3′ downstream (3′-2kb).

## Data Availability

The sRNAome, degradome, transcriptome, and bisulfite sequencing data have been deposited in the Genome Sequence Archive (GSA) at the National Genomics Data Center (NGDC) under the accession number PRJCA014611.

## Supplementary Materials

The following supporting information can be downloaded at: https://www.mdpi.com/article/10.3390/ijms24119150/s1.

## Abbreviations

PH	Plant height
sRNA	Small RNA
miRNA	MicroRNA
siRNA	Small interfering RNA
HPE/LPE	High/Low-parental expressed
TCM/TCdM	Trans-chromosomal methylation/demethylation
NIM	Non-interactive methylation
RdDM	RNA-directed DNA methylation
MPH/BPH	Mid/Better-parent heterosis
DEM/DES/DEG	Differentially expressed miRNA/siRNA/gene
DMR	Differentially methylation region
