# Supplementary material for: Integrated Multi-Omics Reveals Significant Roles of Non-Additively Expressed Small RNAs in Heterosis for Maize Plant Height
Source: Int J Mol Sci. 2023 May 23;24(11):9150. doi: 10.3390/ijms24119150 (PMC10252383; doi:10.3390/ijms24119150)
Supplement: Supplementary file 1 [file ijms-24-09150-s001.zip › F1.sRNA_Supplementary_Figures_Tables_annotation.pdf]

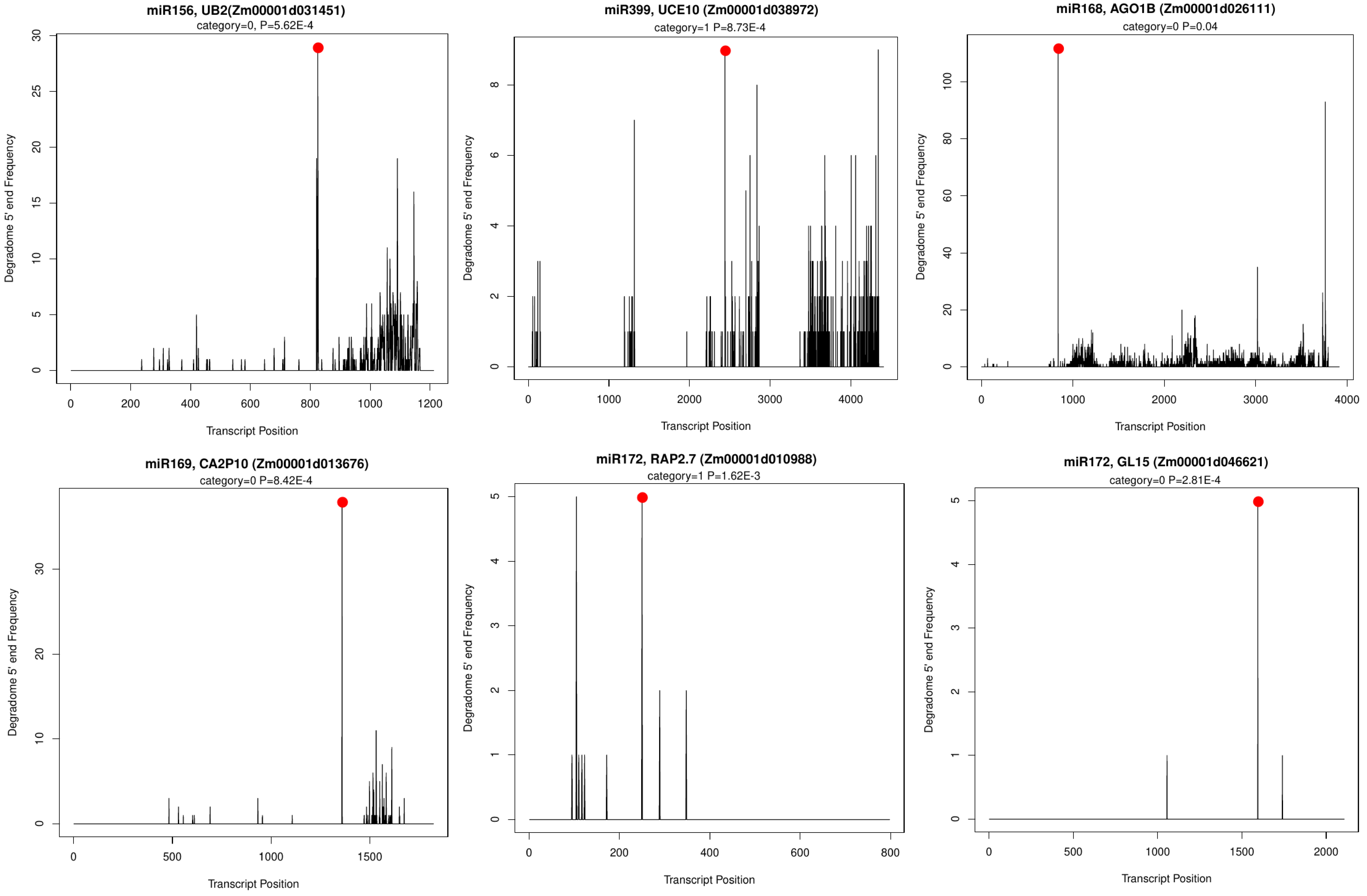

Figure S1. Tplots of target genes cleaved by miRNAs.

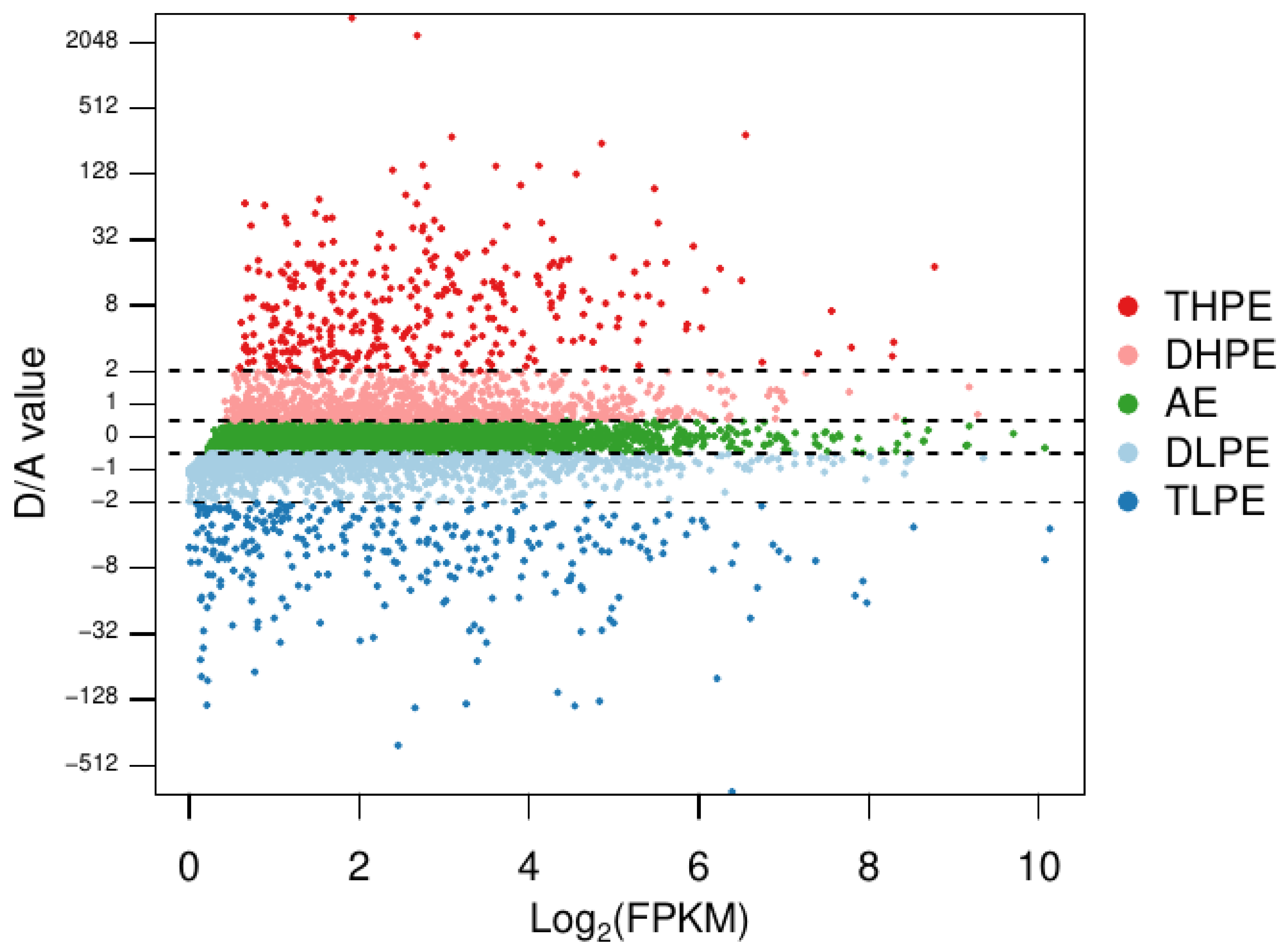

Figure S2. Scatter plot of DEGs of the 5 expression patterns.

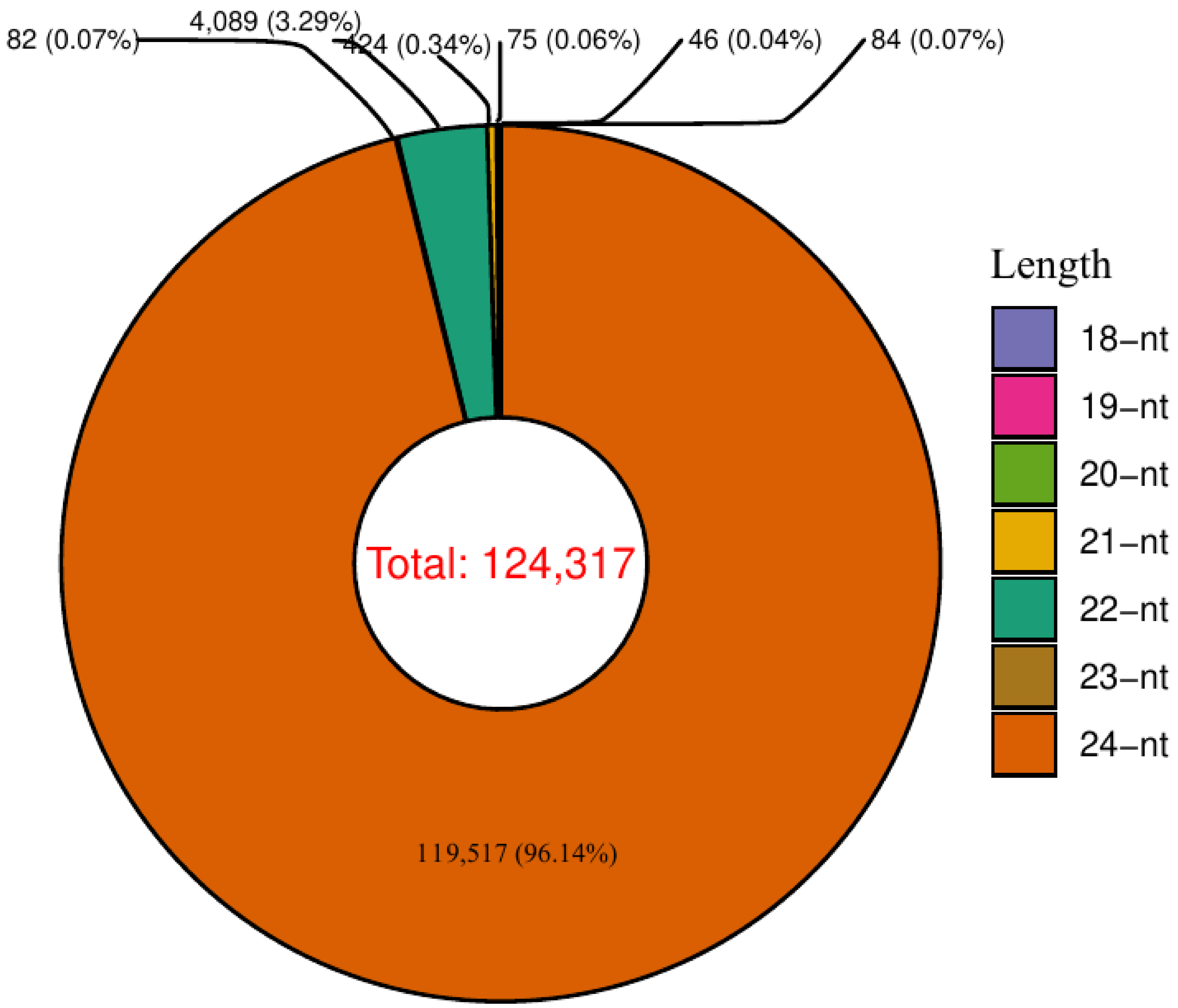

Figure S3. Proportion of 7 types of siRNA clusters identified in the 9 libraries.

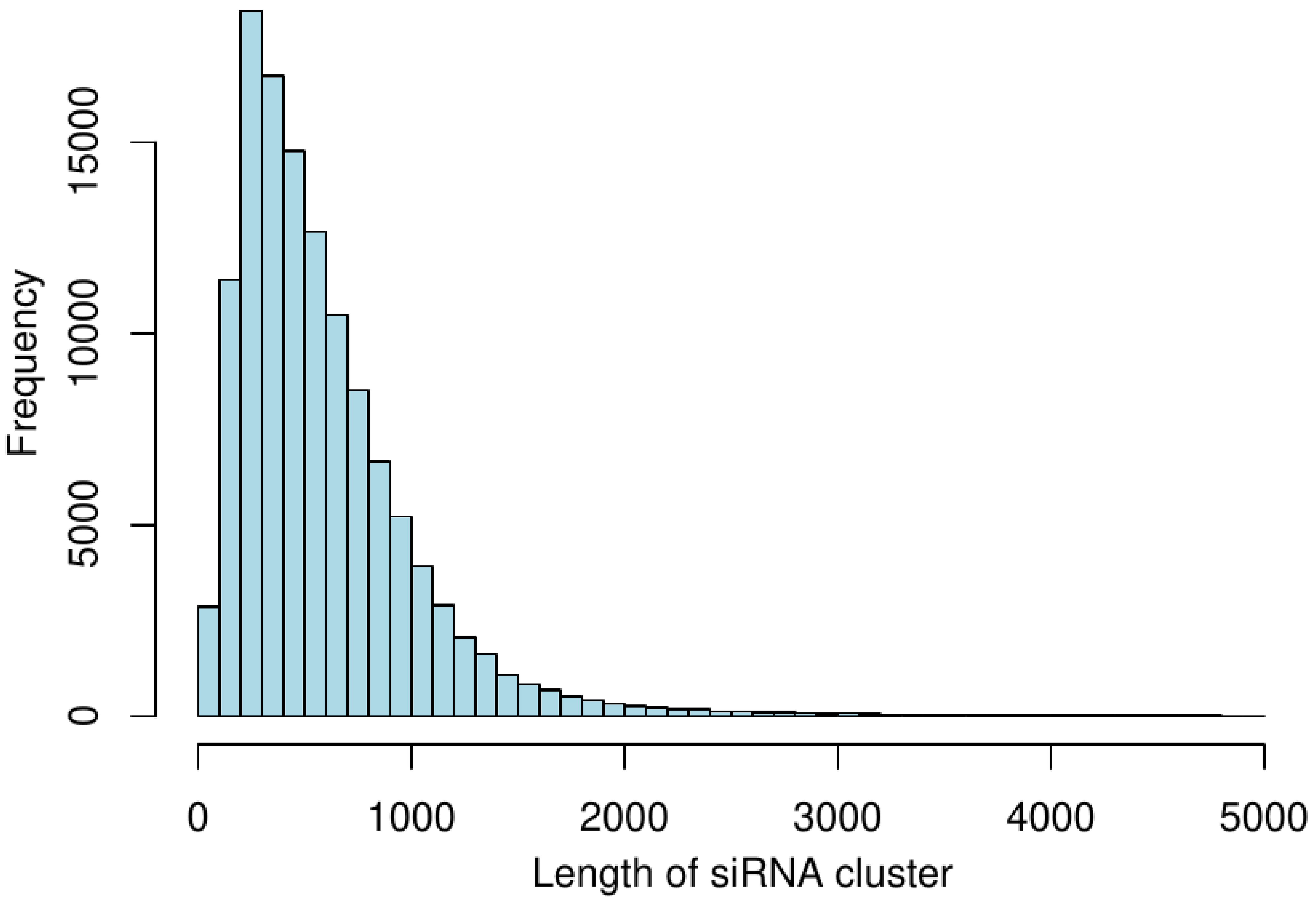

Figure S4. Distribution of the length of siRNA clusters.

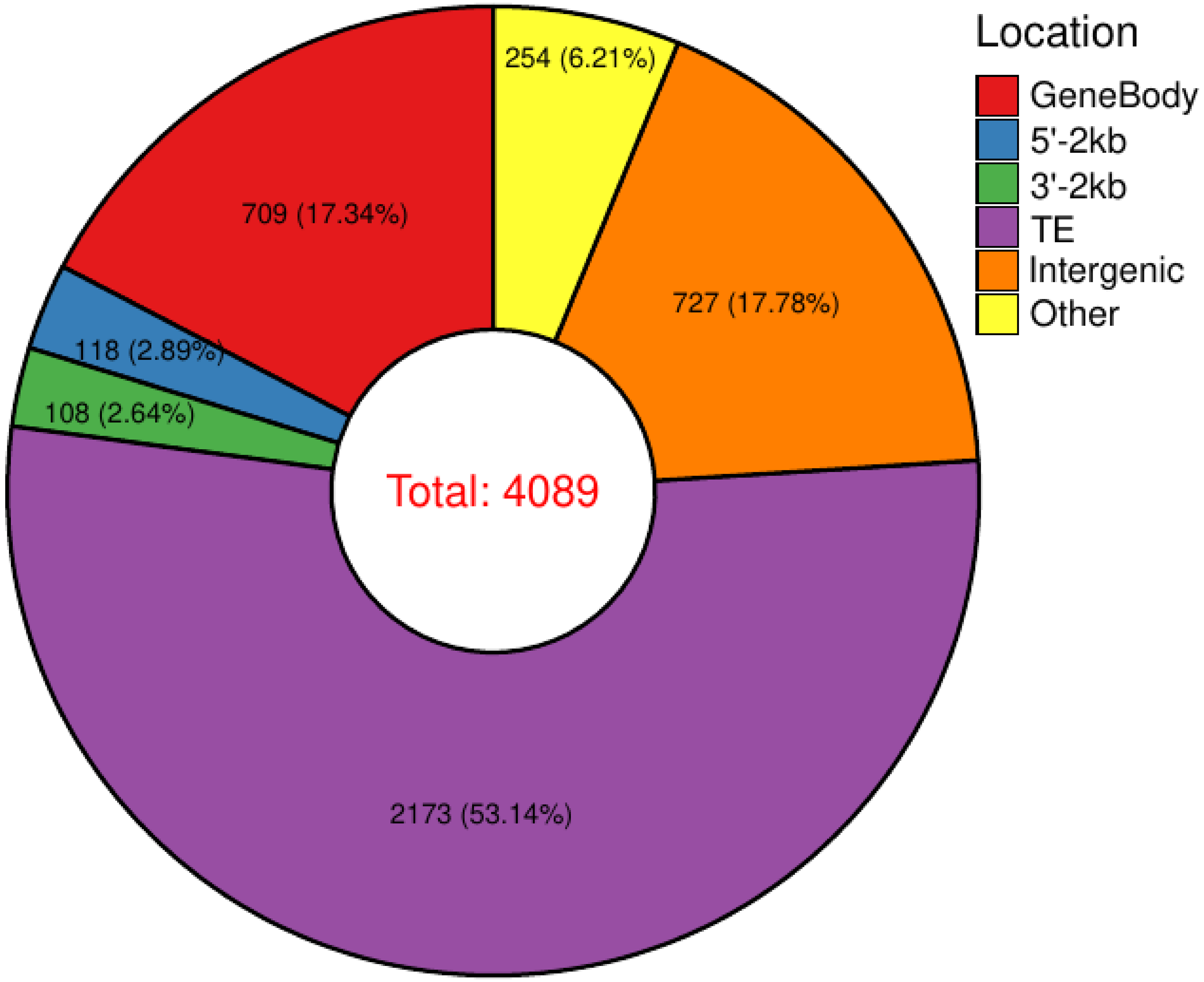

Figure S5. Donut plot of 24-nt siRNA clusters based on the types of genomic feature.

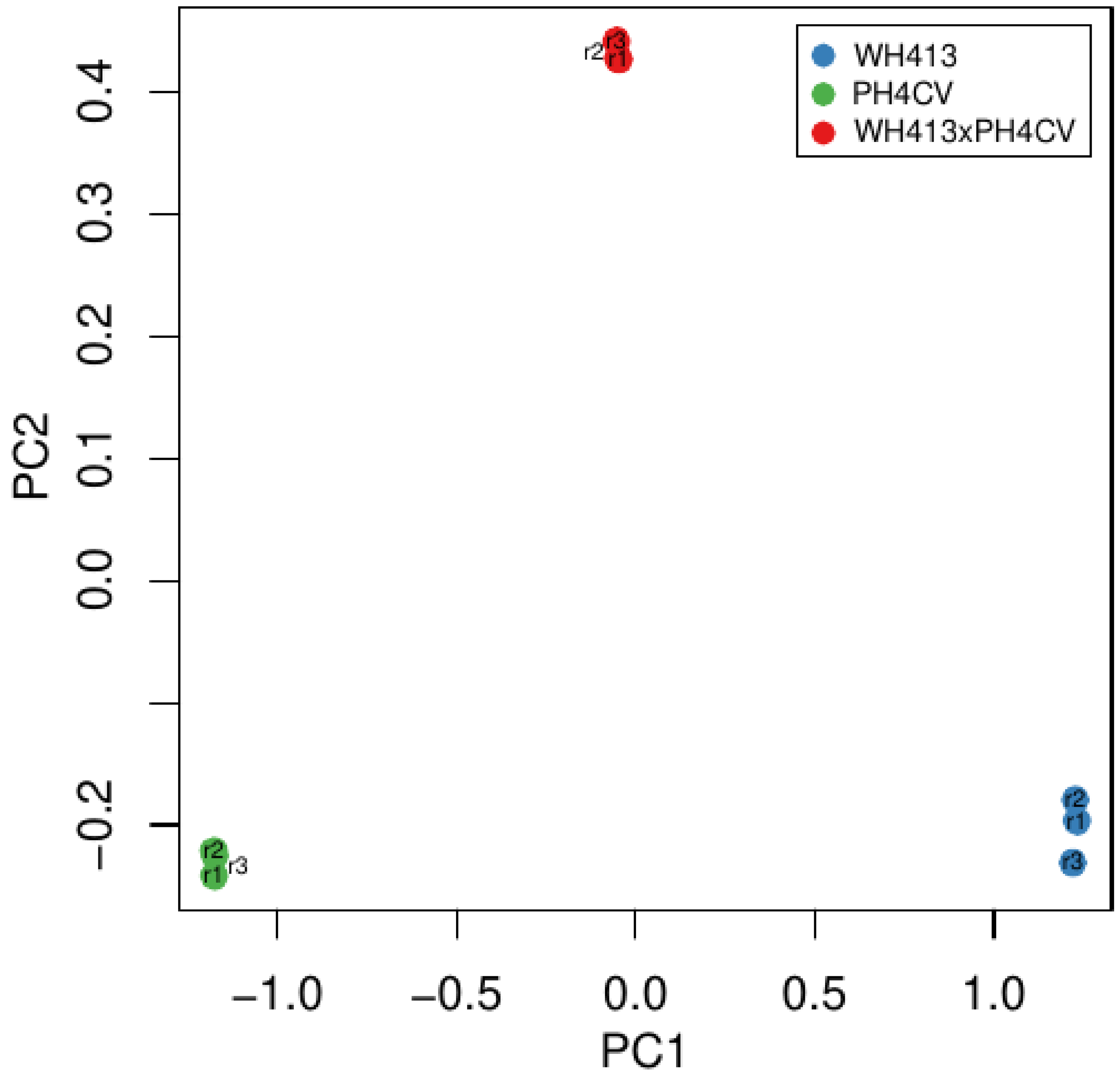

Figure S6. Scatter plot of samples based on PCA of siRNA expression.

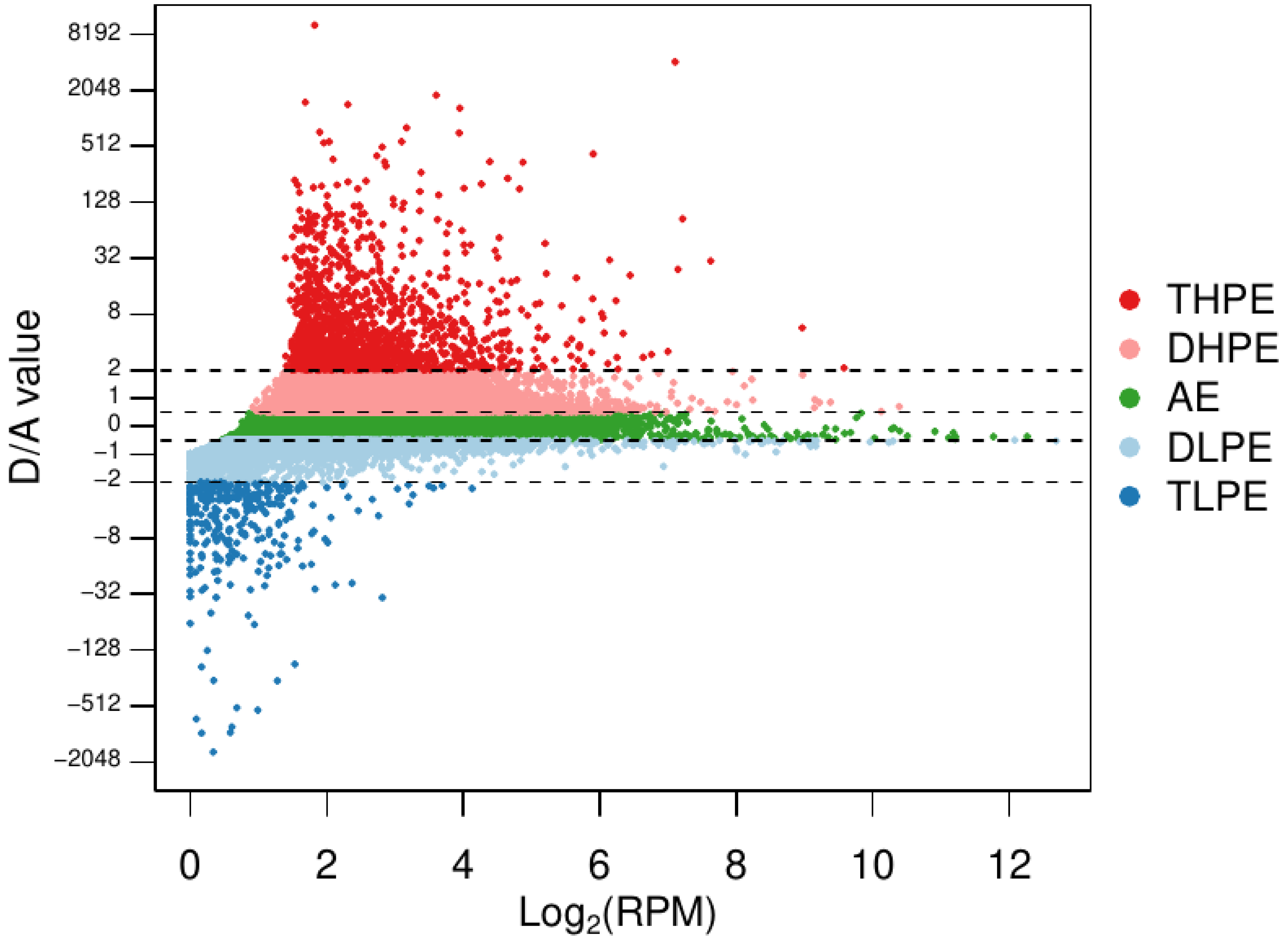

Figure S7. Scatter plot of DESs of the 5 expression patterns

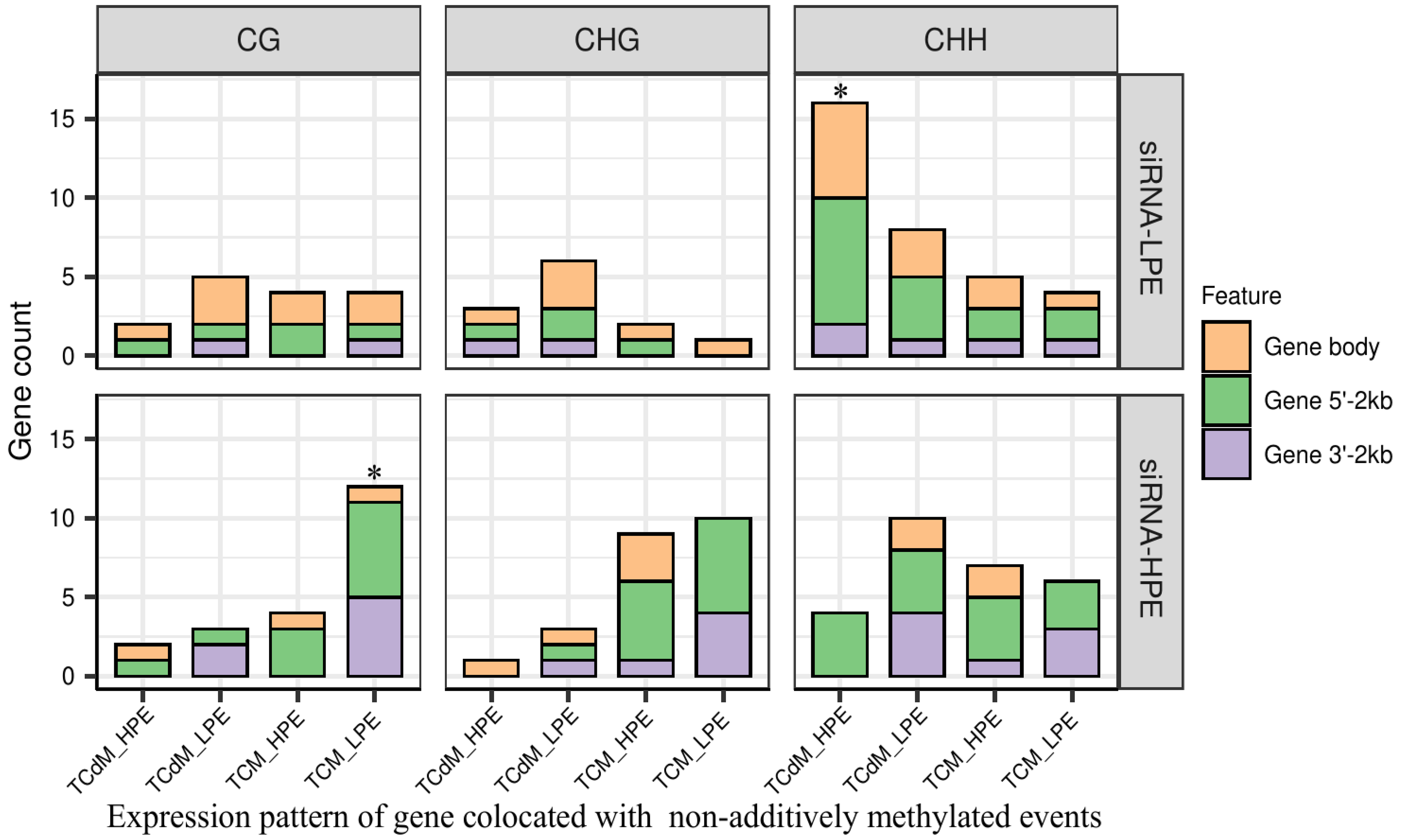

Figure S8. Counts and expression patterns of gene associated with non-additively expressed and methylated siRNA clusters. The single asterisk indicates statistic significance of *P*-value < 0.05.

Table S1. Information of miRNAs identified in the present study.

Table S2. Expression patterns of miRNAs in hybrids.

Table S3. Target genes of miRNAs.

Table S4. Expression patterns of genes in hybrids.

Table S5. siRNA clusters identified by ShortStack.

Table S6. Detail information of siRNAs, including genomic features, expression patterns, and methylation patterns of three sequence contexts.

Table S7. The protein-coding gene-associated siRNA clusters belonging to the six focused groups.
